# The Role of Personality Traits in Young Adult Fruit and Vegetable Consumption

**DOI:** 10.3389/fpsyg.2017.00119

**Published:** 2017-02-07

**Authors:** Tamlin S. Conner, Laura M. Thompson, Rachel L. Knight, Jayde A. M. Flett, Aimee C. Richardson, Kate L. Brookie

**Affiliations:** ^1^Department of Psychology, University of Otago Dunedin, New Zealand; ^2^Department of Psychological Medicine, University of Auckland Auckland, New Zealand

**Keywords:** personality, Mediterranean diet, health behaviors, daily diary methods, diet, young adult

## Abstract

This project investigated how individual differences in the big-five personality traits (neuroticism, extraversion, openness to experience, conscientiousness, and agreeableness) predicted plant-food consumption in young adults. A total of 1073 participants from two samples of young adults aged 17–25 reported their daily servings of fruits, vegetables, and two unhealthy foods for comparison purposes using an Internet daily diary for 21 or 13 days (micro-longitudinal, correlational design). Participants also completed the Neuroticism, Extraversion, Openness Five Factor Inventory (NEO-FFI) measure of personality, and demographic covariates including gender, age, ethnicity, and body mass index (BMI). Analyses used hierarchical regression to predict average daily fruit and vegetable consumption as separate dependent variables from the demographic covariates (step 1) and the five personality traits (step 2). Results showed that young adults higher in openness and extraversion, and to some extent conscientiousness, ate more fruits and vegetables than their less open, less extraverted, and less conscientious peers. Neuroticism and agreeableness were unrelated to fruit and vegetable consumption. These associations were unique to eating fruit and vegetables and mostly did not extend to unhealthy foods tested. Young adult women also ate more fruit and vegetables than young adult men. Results suggest that traits associated with greater intellect, curiosity, and social engagement (openness and extraversion), and to a lesser extent, discipline (conscientiousness) are associated with greater plant-food consumption in this population. Findings reinforce the importance of personality in establishing healthy dietary habits in young adulthood that could translate into better health outcomes later in life.

## The role of personality traits in young adult fruit and vegetable consumption

Fruit and vegetable (FV) consumption is an essential component of a healthy diet that is linked to greater longevity and better physical and mental health (e.g., Liu, 2003; Jacka et al., 2011; Wang et al., 2014; Sánchez-Villegas et al., 2015). Given the importance of this healthy habit, it is important to understand what factors predict higher FV consumption. The majority of research has focused on how demographic factors like gender and BMI predict FV consumption, with evidence that women and lower BMI individuals are more likely to eat fruits and vegetables than men and higher BMI individuals (Biing-Hwan and Morrison, 2002; Gilmour et al., 2010; Konttinen et al., 2010; Heo et al., 2011; Emanuel et al., 2012; Charlton et al., 2014). Less is known about how psychological factors—namely, personality traits—influence FV consumption.

There is growing evidence that personality traits influence a range of health behaviors that can accumulate over the lifespan to impact health and mortality (John and Srivastava, 1999; Friedman, 2000; Bogg and Roberts, 2004; Rhodes and Smith, 2006; Hampson and Friedman, 2008; Taylor et al., 2009; Jokela et al., 2013; Turiano et al., 2015; Dermody et al., 2016). Of the Big Five personality traits, conscientiousness often stands out as a significant predictor of health behaviors (for a meta-analysis see Bogg and Roberts, 2004). For example, lower conscientiousness and higher neuroticism have been shown to predict higher rates of smoking, alcohol use, and lower medication adherence, which can contribute to earlier death (e.g., Friedman, 2000). Recent evidence from the Midlife in the United States (MIDUS) study found that lower conscientiousness predicted higher alcohol use, smoking, and waist circumference, all of which increased the risk of dying 14-years later (Turiano et al., 2015). Lower conscientiousness has also been associated with obesity (Jokela et al., 2013) and lower physical activity (Rhodes and Smith, 2006). Similarly, higher openness has been associated with lower midlife cardiometabolic risk (along with the meta-trait of stability; Dermody et al., 2016), as well as reduced mortality (Taylor et al., 2009), albeit less consistently than conscientiousness, and through unknown behavioral mechanisms (e.g., Taylor et al., 2009; Dermody et al., 2016).

Less is known about how personality traits influence FV consumption, particularly among young adults. This question is important because FV consumption is vital to health and is associated with lower all-cause and cardiovascular mortality (Wang et al., 2014), reduced depression (Jacka et al., 2011; but see Quirk et al., 2013; Sánchez-Villegas et al., 2015), and greater happiness (Blanchflower et al., 2012; White et al., 2013; Mujcic and Oswald, 2016). Moreover, young adults are especially at risk for not meeting the recommended daily intake (RDI) of two servings of fruit and three servings of vegetables each day (Thompson et al., 1999; Guenther et al., 2006; Poortinga, 2007; Kimmons et al., 2009; Krebs-Smith et al., 2010; University of Otago and Ministry of Health, 2011). Studies from the United States, United Kingdom, and New Zealand have found that young adults between 18 and 25 years old have the lowest FV consumption compared to almost all other age groups (Thompson et al., 1999; Poortinga, 2007; Krebs-Smith et al., 2010; University of Otago and Ministry of Health, 2011). These patterns could set them up for poor lifetime habits. Yet some young adults in these surveys are meeting the RDI for fruits and vegetables. These individual differences raise the question of whether psychological factors might explain some of the variation in young adults' FV consumption over and above known demographic characteristics. There is a need for high-quality research testing the link between personality traits and FV consumption in the young adult population.

Among children and adolescents, two personality traits consistently predict higher FV consumption—openness and agreeableness (de Bruijn et al., 2005; Vollrath et al., 2012). In a survey of 327 Norwegian children aged 6–12 years, children higher in imagination (used to measure openness) and benevolence (used to measure agreeableness) were more likely to eat fruit and vegetables as measured by reports from the mothers on a scale from 0 (rarely/never) to 4 (two or more times per day; Vollrath et al., 2012). For boys, extraversion and conscientiousness also predicted increased FV consumption, whereas neuroticism predicted decreased FV consumption (Vollrath et al., 2012). Another survey study of 825 Dutch adolescents aged 12–18 years similarly found that adolescents higher in openness and agreeableness (using a Dutch version of Goldberg's adjective list: Goldberg, 1992; Gerris et al., 1998) reported eating more fruit and vegetables than adolescents low on these traits (de Bruijn et al., 2005). No differences were found for extraversion, conscientiousness, or neuroticism when adjusted for age and gender (de Bruijn et al., 2005). Thus, there seems to be convergent findings among children and adolescents that openness and agreeableness predict higher FV consumption. Openness could reflect a greater willingness to try new foods (e.g., bitter vegetables) that might not be immediately palatable in young age, whereas agreeableness may reflect a greater likelihood to eat what parents ask them to eat, including fruit and vegetables (de Bruijn et al., 2005).

In older adult populations, openness is a key predictor of FV consumption. In a study by Tiainen et al. (2013), 1681 Finnish older adults (mean age = 61.5 years) reported their personality traits using the NEO-Five Factor Inventory (NEO-FFI; Costa and McCrae, 1992). Several traits were associated with FV consumption, but openness was the most consistent (Tiainen et al., 2013). In women, higher openness was associated with greater FV consumption; in men, higher openness was associated with increased vegetable but not fruit consumption (Tiainen et al., 2013). Another study of 951 adults (mean age = 55 years old) tested the relationship between personality, eating styles, and food choices (Keller and Siegrist, 2015). Again, openness showed a direct relationship to FV consumption, with adults higher in openness reporting a greater intake of fruit and vegetables. Other personality factors were found to influence FV consumption indirectly through differences in eating styles (e.g., conscientiousness predicted restrained eating, which predicted greater fruit consumption). Unlike the previously mentioned studies of children and adolescents, the studies of adult populations typically show no or few associations between agreeableness and FV consumption (Keller and Siegrist, 2015). Therefore, the findings for openness appear consistent across age groups, whereas agreeableness differs across age groups. Somewhere between adolescence and adulthood, agreeableness stops predicting FV consumption. One possibility is that during the period of young adulthood, agreeableness drops out as predictor of FV consumption because healthy food choices are less parent-driven.

Very little is known about how personality predicts FV consumption among young adults. Young adults in the 18–25 years age-range are an important population to study because they are typically moving out of their childhood home and living on their own in apartments or dormitories. It is often a period of exploration and emerging autonomy over their lifestyle choices compared to children and adolescents (Arnett, 2000). Importantly, the healthy habits young adults establish during this exploratory phase of early adulthood could carry through to later adulthood, ultimately affecting health throughout the lifespan. We found only one study of young adults by Raynor and Levine (2009) who surveyed 603 university students and tested the relationship between personality traits and seven preventive health behaviors including typical FV consumption assessed by a single question “How many servings of fruits and vegetables do you usually have per day?” Their results showed that higher conscientiousness and higher openness predicted greater FV consumption. Furthermore, whereas conscientiousness predicted higher rates of all preventive health behaviors (seat belt use, exercise, sleep, less alcohol use, condom use, and higher FV consumption), openness only predicted higher FV consumption. This finding suggests that openness has a unique relationship to FV consumption. However, that study was limited by the measurement of FV consumption and the lack of demographic control variables such as gender and BMI. Not controlling for gender could confound relationships because women eat more FV than men (e.g., Konttinen et al., 2010; Emanuel et al., 2012) and women score higher in neuroticism, openness, and agreeableness than men (e.g., Weisberg et al., 2011). Similarly, lower BMI is associated with higher fruit and vegetable consumption (Biing-Hwan and Morrison, 2002; Heo et al., 2011) and there is evidence that BMI is positively associated with personality traits such as neuroticism and extraversion, and inversely associated with conscientiousness (Sutin et al., 2011).

The aim of the current study was to test the relationship between the big-five personality traits and daily self-reported FV consumption among a large population of young adults ages 17–25. We used two data sets totaling 1073 participants. Participants reported their consumption of fruits and vegetables at the end of each day (and two unhealthy foods for comparison purposes) for either 21 or 13 days using an Internet daily diary procedure. They also completed a standard measure of the Big Five personality traits (Neuroticism, Extraversion, and Openness Five Factor Inventory, NEO-FFI; Costa and McCrae, 1992) and demographic characteristics including gender, age, ethnicity, and BMI as control variables. Gender and BMI were included as control variables given previous research linking these factors to both FV consumption and personality. Age was included as a continuous control variable due to evidence that fruit and vegetable consumption increases across the young adult years even within a narrow 17–25 age range (Guenther et al., 2006; Kimmons et al., 2009). In summary, the use of two large replication samples combined with intensive daily tracking of FV consumption and proper control variables allowed for a strong test of the association between personality and FV consumption in this young adult age group.

We hypothesized that openness to experience would be the strongest predictor of greater FV consumption because this trait consistently predicts FV in other age groups and there is good theoretical justification for this link (i.e., openness is characterized by higher intellect, greater curiosity, and a willingness to try new things, which could include initially unpalatable plant-foods; DeYoung, 2010). We also hypothesized that conscientiousness would predict higher FV consumption, albeit to a lesser extent than openness given the inconsistencies in previous research findings with regards to FV. Lastly, we hypothesized that agreeableness would not predict young adult FV consumption because young adults are making their own food choices and are not under parental control. We also tested for gender differences in the association between personality traits and FV consumption because some previous research found gender differences. However, we made no hypotheses because of the inconsistency with previous findings and the lack of theoretical justification concerning gender differences. These tests were exploratory.

## Methods

### Participants

Table 1 presents the participant characteristics of the two samples. All participants were enrolled as full- or part-time students at the University of Otago, a public New Zealand university of approximately 16,000 undergraduate students. Participants were recruited either as part of their undergraduate psychology course and remunerated with course credits (73.7% of Sample 1; 57.4% of Sample 2), or through a student employment agency, classes, or word of mouth and were remunerated with a cash payment up to NZD$70 for Sample 1 or NZD$55 for Sample 2. Each study was broadly advertised as a “study of the daily lives of university students” with no reference to the specific aims. The specific aims were written only on an information sheet that accompanied the consent form (Sample 1 “to understand what happens in the daily lives of Otago students.” Sample 2 “to understand how genes, hormones, and other biomarkers like Vitamin D and iron are related to daily well-being among university students.”) The hypotheses tested in this article fell under the umbrella aim to understand the health and well-being of young adults. In Sample 1, we recruited additional male students through the student employment agency to balance out the gender distribution. In Sample 2, we recruited both men and women through the student employment agency, nutrition and medicine classes, and flyers in addition to recruitment from psychology courses. Thus, Sample 2 was more heterogeneous than Sample 1. Recruitment and testing were conducted between April 2008 and August 2009 for Sample 1 and between April 2013 and August 2014 for Sample 2. As shown in Table 1, the majority of participants in both samples were of European ancestry. The ethnicity distribution for Sample 1 was 235 (83.6%) European/Caucasian, 15 (5.3%) Asian, 11 (3.9%) Māori/Pacific Island, 4 (1.4%) Indian, and 16 (5.7%) of another or mixed ethnicity. The ethnicity distribution for Sample 2 was similar with 614 (77.5%) European/Caucasian, 86 (10.9%) Asian, 43 (5.4%) Māori/Pacific Island, 29 (3.7%) Indian, and 20 (2.5%) of another or mixed ethnicity. These ethnic distributions matched the wider university population.

**Table 1:** **Participant characteristics and descriptive statistics for the two samples of young adults**.

	**Mean**	***SD***	**Minimum**	**Maximum**	***n* (%)**
**SAMPLE 1**					
N					281
Male					128 (45.6%)
Female					153 (54.4%)
% European					83.6%
Age (years)	19.90	1.24	17.00	25.00	
BMI^a^	23.78	3.47	16.18	37.78	
Neuroticism	2.80	0.73	1.25	4.50	
Extraversion	3.51	0.50	1.58	4.83	
Openness	3.49	0.50	2.17	4.83	
Conscientiousness	3.32	0.60	1.75	4.83	
Agreeableness	3.59	0.47	2.17	4.75	
Fruit/day	1.70	1.08	0.00	5.73	
Vegetables/day	2.51	1.07	0.20	5.76	
Chips/day	0.45	0.57	0.00	5.77	
Cookies/day	0.40	0.42	0.00	2.22	
**SAMPLE 2**					
N					792
Male					217 (27.4%)
Female					575 (72.6%)
% European					77.5%
Age (years)	19.73	1.73	17.00	25.00	
BMI^b^	23.99	4.52	13.43	57.24	
Neuroticism	2.91	0.72	1.08	4.83	
Extraversion	3.51	0.52	1.50	4.83	
Openness	3.46	0.53	2.08	4.75	
Conscientiousness	3.49	0.61	1.25	4.92	
Agreeableness	3.74	0.50	2.25	4.83	
Fruit/day	2.08	1.29	0.00	7.00	
Vegetables/day	2.76	1.38	0.00	7.70	
Fries/day	0.56	0.60	0.00	4.08	
Candy/day	1.27	0.94	0.00	6.30	

BMI, body mass index.

a BMI computed from self-reported height and weight.

b *BMI computed from objectively measured height and weight. Food consumption variables expressed in standard serving sizes*.

### Procedure

In both samples, participants attended an initial laboratory visit where they were briefed on the study and completed an initial survey by computer. The survey included measures of personality and demographics including gender, age, and ethnicity, embedded within several other measures not relevant to the present report. In Sample 1, the initial survey also included self-reported height and weight used to compute BMI. Following this initial laboratory visit, participants completed an Internet-based daily diary procedure for either 21 consecutive days starting the following Monday (Sample 1) or for 13 days starting the following day to accommodate more participants in a shorter time span (Sample 2). The daily diary procedure was shortened by a week in Sample 2 to reduce participant burden without sacrificing sensitivity to detect typical dietary patterns (Conner and Lehman, 2012). The daily diary was accessed through a password-protected website between 3 and 8 p.m. and took approximately 5 min to answer. Participants received an evening reminder via text message to complete the survey if they had not done so already. After the 21 or 13 days of tracking, participants attended a second laboratory session for a follow up survey containing additional measures not relevant to this report. Participants in Sample 2 also attended a clinic on the last day of the study, during which height and weight was measured by a trained anthropometrist to compute BMI.

### Measures

#### Personality

In both samples, the Big Five personality traits were assessed using the 60-item Neuroticism, Extraversion, Openness Five Factor Inventory (NEO-FFI; Costa and McCrae, 1992). There were 12 items for each of the five traits. For example, neuroticism consisted of items such as “*I often feel inferior to others*” and “*I often feel tense or jittery*.” Extraversion included items such as “*I laugh easily*” and “*I really enjoy talking to people;*” Openness, “*I have a lot of intellectual curiosity*.” and “*I enjoy playing with theories or abstract ideas;*” Conscientiousness, “*I strive for excellence in everything I do*” and “*Sometimes I'm not as dependable or reliable as I should be*” (reversed scored); and, agreeableness “*Most people I know like me*” and “*I would rather cooperate with others than compete with them*.” Participants rated each of these statements on a 5-point Likert scale from 1 (*strongly disagree)* to 5 (*strongly agree*). Personality scores were calculated by averaging across the 12 items (reverse scoring certain items) for separate measures of neuroticism (Sample 1 and 2 α = 0.88; 0.86), extraversion (α = 0.76; 0.80), openness (α = 0.71; 0.74), conscientiousness (α = 0.85; 0.86), and agreeableness (α = 0.73; 0.76).

#### Fruit and vegetable consumption

Participants completed daily diaries that included measures of their fruit and vegetable consumption from the previous night (i.e., “*In the time between completing yesterday's survey and going to bed”*), and separately, for that day (i.e., “*From the time you woke up until now”*) using two items modified from the New Zealand National Nutrition Survey 1997 (NNS'97; Russell et al., 1999). Specifically, participants were asked, “*How many servings of fruit (fresh, frozen, canned or stewed) did you eat today? Do not include fruit juice or dried fruit,”* and for vegetables, “*How many servings of vegetables (fresh, frozen, canned, or stewed) did you eat today? Do not include vegetable juices or hot chips (French fries).”* Examples of serving size estimates were provided in order to ensure participants had accurate and consistent self-report [e.g., *1 serving of fruit equals 1 medium piece of fruit (e.g., apple) or 2 small pieces of fruit (e.g., 2 apricots); 1 serving of vegetables equals 1 medium potato*, ½ *cup cooked vegetables, or 1 cup of salad*.] Participants in Sample 1 reported their number of servings using a response scale of 0–3 (0 = no servings, 1 = 1 serving, 2 = 2 servings, 3 = 3 or more servings). Participants in Sample 2 used an expanded response scale of 0–4 (0 = no servings; 0.5 = <1 serving, 1 = 1 serving, 2 = 2 servings, 3 = 3 servings, 4 = 4 or more servings). In previous research, we found these items sensitive for measuring mean differences and intra-individual variation in FV consumption (White et al., 2013).

#### Unhealthy food consumption

For comparison purposes, participants also reported their previous night and daytime consumption of a savory and sweet unhealthy food in the daily diary. Items were modified from the New Zealand National Nutrition Survey 1997 (NNS'97; Russell et al., 1999). Participants in Sample 1 reported their consumption of potato chips [“*How many servings of crisps (potato chips), corn snacks or corn chips did you eat today?” (0* = *none, to 4* = *4 or more) where 1 serving equals 1 small packet (40–45 g) and 4 servings equals 1 large packet*] and cookies [“*How many chocolate coated and/or cream filled biscuits (cookies) did you eat today?” (0* = *none, 1* = *1–2 biscuits, 2* = *3–4 biscuits, 3* = *5 or more biscuits)*]. Participants in Sample 2 reported their consumption of fries [“*How many servings of hot chips, French fries, wedges, or sweet potato chips did you eat today?*” *(0* = *none, to 4* = *4 or more) where 1 serving equals one cup or 1 small/regular fast food serving and 2 servings equals one large fast food serving*] and candy [*How many servings of lollies (candy), sweets, chocolate, or other confectionary items did you eat today? (0* = *none, to 4* = *4 or more) where 1 serving equals one regular sized chocolate bar (*~*50 g) or the amount that would fit into the palm of your hand*].

### Data preparation and analysis

Exclusion criteria for this study were: completion of fewer than 11 diaries for Sample 1 (*n* = 6); completion of fewer than seven diaries for Sample 2 (*n* = 31); or unavailable BMI data in Sample 2 (*n* = 4). Those who did not meet the inclusion criteria were excluded from analyses. Excluded participants in Sample 1 were more likely to score higher in openness than included participants [4.1 vs. 3.5; *t*_(285)_ = 2.95, *p* = 0.003]. Excluded participants in Sample 2 were more likely to be male [χ^2^_(1,*N*=827)_ = 7.41, p = 0.007], and score lower in conscientiousness [3.1 vs. 3.5; *t*_(825)_ = −3.58, *p* < 0.001] and agreeableness [3.5 vs. 3.7; *t*_(825)_ = −3.35, *p* = 0.001] than included participants. This left a total of 281 participants in Sample 1 who completed on average 19 out of 21 daily diaries (*SD* = 2, range = 11–21; 91% compliance) and a total of 792 participants in Sample 2 who completed on average 12 out of 13 daily diaries (*SD* = 2, range = 7–13; 89% compliance). In order to represent food consumption for a full day, the day- and night-time food serving reports were added together for a measure of total daily consumption of each food variable. Days with only one entry (day- or night-time) were removed from analysis, which reduced the number of analyzed diary records in Sample 1 to an average of 16 out of 21 daily diaries (76% compliance) totaling 4,593 daily records, and in Sample 2, to an average 10 out of 13 (73% compliance) totaling 7,548 daily records. Missing diaries were randomly distributed across the days of week in Sample 1 [χ^2^_(6, *N* = 5320)_ = 8.068, *p* = 0.234] and more likely to be missing on weekdays than weekends in Sample 2 [χ^2^_(6, *N* = 10,349)_ = 26.28, *p* < 0.001]. Thus, there were more weekends days represented in the diary data of Sample 2. Little's (1988) Missing Completely at Random tests (MCAR) of the four daily diary variables revealed a pattern of missingness not completely random in Sample 1 [$\chi_{\left(18\right)}^{2}$ = 35.41, *p* = 0.008] but completely random in Sample 2 [$\chi_{\left(21\right)}^{2}$ = 22.40, *p* = 0.377]. Follow-up tests of Sample 1 showed few relationships between the five personality traits and the frequency of missing data, with one exception: Higher conscientiousness was related to fewer missing data on all four diary variables (*r*s ranging from −0.214 to −0.225, all *p*s < 0.001). This pattern was due to conscientious participants completing, on average, 1 diary record more than low conscientious participants (19.4 vs. 18.5 diary records for participants +1 vs. −1 *SD* in conscientiousness, *r* = 0.251, *p* < 0.001). However, we did not perform any missing diary data imputation given the small difference between completed diary records for low vs. high conscientious participants in Sample 1.

Prior to analysis, each person's average daily consumption of fruit, vegetables, and the two unhealthy foods were averaged across the diaries to create four aggregated outcome variables (average daily consumption of fruit, vegetables, chips/fries, and cookies/candy). For analyses, hierarchical multiple regression was used with average daily fruit and vegetable consumption, and the two unhealthy foods, as separate dependent variables. Hierarchical multiple regression was chosen over a multilevel modeling approach for parsimony given that the outcome was a simple aggregated measure and both approaches yielded similar patterns. In the first step of the hierarchical regression, the covariates of gender (coded 0 for men, 1 for women), age (centered), BMI (centered), and recruitment pool (coded 0 for Psychology class recruitment, 1 for other recruitment) were entered simultaneously to predict the outcome variable. Recruitment pool was not included as a control variable in the Sample 1 analysis because of multicolinearity with the gender variable. Ethnicity was not included as a covariate because it was unrelated to the food consumption variables. Diary start date (Tues to Sat) was tested as a covariate in the Sample 2 analysis but did not affect results and was removed from the final covariate list. In the second step, the five personality measures (neuroticism, extraversion, openness, conscientiousness, and agreeableness, all standardized) were entered as simultaneous predictors to determine which personality traits uniquely predicted FV consumption over and above the demographic characteristics. In the third step, the cross-product interaction terms between gender and each of the five standardized personality measures were entered as simultaneous predictors. Significant interactions were probed to determine the pattern for men and women separately. *R*-squared change statistics served as the measure of effect size. The continuous measures of age and BMI were centered to make the intercepts more interpretable. The personality measures were standardized to make the intercepts and coefficients more interpretable (i.e., the coefficient reflected the difference in average daily food servings for 1 standard deviation difference in personality).

## Results

### Descriptive statistics

Table 1 shows the descriptive statistics for both samples. The average BMI of participants in both samples was 23.8 and 24.0, respectively, both of which fell within the normal range (18.5–24.9 BMI), however, participants ranged from under-weight (<18.5 BMI) to obese (>29.9 BMI; Heart Foundation, n.d.). The BMI averages were similar to norms for this age group in other countries (Dodd et al., 2010). There was a wider BMI range in the larger and more diverse Sample 2, which included two people classified within the severe thinness range (<16.0 BMI) and seven people classified in the highest obesity class (Obesity Class III, >40.0 BMI). The personality score averages were similar to norms for this age group in other countries (Neyer and Asendorpf, 2001; Robins et al., 2001). Personality scores varied greatly between participants, spanning almost the entire 1–5 range. On average, daily FV consumption was close to the recommended level of five combined servings per day, which is higher than norms for this age range (Unüsan, 2004; Dodd et al., 2010); however, there was wide variation in the average daily servings of fruit and vegetables consumed, from people who consumed 0 servings to 6 or 7 servings per day. Fruit and vegetable consumption was slightly higher in Sample 2, however this may have been because of sample differences (i.e., more participants recruited from nutrition and medicine classes) or because Sample 2 included one higher response category of “4 or more servings” compared to Sample 1. This difference was not due to an overrepresentation of weekend days in Sample 2 because young adults in that Sample 2 ate *fewer* servings of fruit and vegetables on weekend days (Sat–Sun: 4.5 combined daily servings of fruit and vegetables; Mon–Fri: 5.0 combined daily servings of fruit and vegetables).

### Regression results

Tables 2, 3 present the regression results for each sample. The results from Model 2 showed that, across both samples, openness to experience was the most consistent significant predictor of higher fruit and vegetable consumption. Young adults higher in openness ate more daily servings of fruit and vegetables than young adults lower in openness across both samples tested. In terms of serving sizes, young adults one standard deviation above the mean (+1 *SD*) in openness ate 0.26 and 0.26 more daily servings of fruit (Sample 1 and 2, respectively) and 0.38 and 0.36 more daily servings of vegetables (Sample 1 and 2, respectively) compared to participants one standard deviation below the mean (−1 *SD*) in openness. The associations were slightly stronger for vegetable than fruit consumption.

**Table 2:** **Results of hierarchical regression analyses predicting young adults' fruit and vegetable consumption and unhealthy foods in Sample 1 (***N*** = 281)**.

	**Fruit**	**Vegetables**	**Potato chips**	**Cookies**
**MODEL 1**				
Intercept	1.49 (0.10)^***^	2.33 (0.10)^***^	0.52 (0.05)^***^	0.38 (0.04)^***^
Gender	0.39 (0.14)^**^	0.33 (0.14)^*^	−0.12 (0.07)^+^	0.04 (0.05)
Age	0.02 (0.05)	0.09 (0.05)^+^	−0.03 (0.03)	0.01 (0.02)
BMI	0.02 (0.02)	0.00 (0.02)	0.00 (0.01)	−0.01 (0.01)
*R*-square Δ (*df* 1, 2)	0.03 (3, 277)^*^	0.03 (3, 277)^+^	0.01 (3, 277)	0.01 (3, 277)
**MODEL 2**				
Intercept	1.47 (0.10)^***^	2.27 (0.10)^***^	0.53 (0.05)^***^	0.39 (0.04)^***^
Gender	0.42 (0.14)^**^	0.42 (0.14)^**^	−0.14 (0.08)^+^	0.02 (0.06)
Age	0.02 (0.05)	0.09 (0.05)^+^	−0.02 (0.03)	0.01 (0.02)
BMI	0.02 (0.02)	0.01 (0.02)	0.00 (0.01)	−0.01 (0.01)
Neuroticism	0.01 (0.07)	−0.03 (0.07)	0.05 (0.04)	0.07 (0.03)^*^
Extraversion	0.17 (0.07)^*^	0.13 (0.07)^+^	0.03 (0.04)	0.06 (0.03)^*^
Openness	0.13 (0.06)^*^	0.19 (0.06)^**^	−0.10 (0.03)^**^	−0.05 (0.03)^+^
Conscientiousness	0.11 (0.07)^+^	−0.04 (0.07)	−0.05 (0.04)	−0.02 (0.03)
Agreeableness	−0.05 (0.07)	0.02 (0.07)	−0.04 (0.04)	−0.01 (0.03)
*R*-square Δ (*df* 1, 2)	0.05 (5, 272)^*^	0.05 (5, 272)^*^	0.06 (5, 272)^**^	0.04 (5, 272)^*^
**MODEL 3**				
Intercept	1.45 (0.11)^***^	2.28 (0.11)^***^	0.54 (0.06)^***^	0.37 (0.04)^***^
Gender	0.44 (0.14)^**^	0.43 (0.14)^**^	−0.14 (0.08)^+^	0.03 (0.06)
Age	0.03 (0.05)	0.09 (0.05)^+^	−0.03 (0.03)	0.02 (0.02)
BMI	0.03 (0.02)	0.01 (0.02)	0.00 (0.01)	−0.01 (0.01)
Neuroticism	−0.06 (0.11)	−0.03 (0.11)	0.11 (0.06)^+^	0.04 (0.04)
Extraversion	0.17 (0.11)	0.16 (0.11)	0.07 (0.06)	0.07 (0.04)
Openness	0.10 (0.09	0.14 (0.09)	−0.12 (0.05)^*^	−0.03 (0.04)
Conscientiousness	0.10 (0.10)	−0.02 (0.10)	−0.03 (0.05)	−0.03 (0.04)
Agreeableness	−0.16 (0.10)	−0.06 (0.10)	−0.09 (0.05)	−0.03 (0.04)
Gender × Neuro	0.14 (0.15)	−0.01 (0.15)	−0.11 (0.08)	0.05 (0.06)
Gender × Extra	0.01 (0.14)	−0.06 (0.14)	−0.08 (0.08)	−0.01 (0.06)
Gender × Open	0.07 (0.13)	0.09 (0.13)	0.03 (0.07)	−0.04 (0.05)
Gender × Consc	0.02 (0.13)	−0.04 (0.13)	−0.03 (0.07)	0.02 (0.05)
Gender × Agree	0.18 (0.14)	0.16 (0.14)	0.09 (0.07)	0.05 (0.05)
*R*-square Δ (*df* 1, 2)	0.01 (5, 267)	0.01 (5, 267)	0.02 (5, 267)	0.01 (5, 267)

*BMI, body mass index; Δ, change. Numbers reflect unstandardized regression coefficients (with standard errors). Gender was uncentered (0 = men, 1 = women). Age and BMI were centered. Neuroticism, Extraversion, Openness, Conscientiousness, and Agreeableness were standardized. The intercept reflects the average number of daily servings for men at mean levels on the other predictor variables*.

+ p < 0.10;

* p < 0.05;

** p < 0.01;

*** *p < 0.001*.

**Table 3:** **Results of hierarchical regression analyses predicting young adults' fruit and vegetable consumption and unhealthy foods in Sample 2 (***N*** = 792)**.

	**Fruit**	**Vegetables**	**Fries**	**Candy**
**MODEL 1**				
Intercept	1.83 (0.10)^***^	2.34 (0.11)^***^	0.82 (0.05)^***^	0.96 (0.07)^***^
Gender	0.15 (0.10)	0.36 (0.11)^**^	−0.27 (0.05)^***^	0.36 (0.07)^***^
Age	−0.08 (0.03)^**^	−0.06 (0.03)^+^	−0.01 (0.01)	−0.02 (0.02)
BMI	−0.01 (0.01)	−0.01 (0.01)	0.00 (0.00)	0.01 (0.01)
Recruitment	0.34 (0.10)^**^	0.38 (0.11)^***^	−0.16 (0.05)^**^	0.13 (0.07)^+^
*R*-square Δ (*df* 1, 2)	0.02 (4, 787)^**^	0.03 (4, 787)^***^	0.06 (4, 787)^***^	0.03 (4, 787)^***^
**MODEL 2**				
Intercept	1.84 (0.10)^***^	2.37 (0.11)^***^	0.79 (0.05)^***^	0.90 (0.08)^***^
Gender	0.14 (0.11)	0.33 (0.11)^**^	−0.23 (0.05)^***^	0.42 (0.08)^***^
Age	−0.09 (0.03)^**^	−0.07 (0.03)^*^	−0.01 (0.01)	−0.02 (0.02)
BMI	0.00 (0.01)	0.00 (0.01)	0.00 (0.00)	0.00 (0.01)
Recruitment	0.33 (0.10)^**^	0.35 (0.10)^**^	−0.14 (0.05)^**^	0.15 (0.07)^*^
Neuroticism	0.02 (0.05)	−0.02 (0.06)	0.02 (0.02)	0.01 (0.04)
Extraversion	0.26 (0.05)^***^	0.20 (0.05)^***^	0.04 (0.02)^+^	0.02 (0.04)
Openness	0.11 (0.05)^*^	0.19 (0.05)^***^	−0.08 (0.02)^***^	−0.08 (0.04)^*^
Conscientiousness	0.13 (0.05)^**^	0.18 (0.05)^***^	−0.03 (0.02)	0.00 (0.03)
Agreeableness	−0.06 (0.05)	0.00 (0.05)	−0.08 (0.02)^**^	−0.12 (0.04)^**^
*R*-square Δ (*df* 1, 2)	0.05 (5, 782)^***^	0.06 (5, 782)^***^	0.03 (5, 782)^***^	0.02 (5, 782)^**^
**MODEL 3**				
Intercept	1.84 (0.11)^***^	2.31 (0.12)^***^	0.85 (0.05)^***^	0.91 (0.08)^***^
Gender	0.14 (0.11)	0.39 (0.12)^**^	−0.29 (0.05)^***^	0.41 (0.08)^***^
Age	−0.09 (0.03)^**^	−0.07 (0.03)^*^	−0.01 (0.01)	−0.02 (0.02)
BMI	−0.01 (0.01)	0.00 (0.01)	0.00 (0.00)	0.00 (0.01)
Recruitment	0.33 (0.10)^**^	0.34 (0.10)^**^	−0.13 (0.05)^**^	0.16 (0.07)^*^
Neuroticism	0.00 (0.10)	−0.18 (0.10)^+^	0.14 (0.05)^**^	0.02 (0.07)
Extraversion	0.38 (0.10)^***^	0.13 (0.10)	0.08 (0.05)^+^	−0.04 (0.07)
Openness	0.10 (0.09)	0.14 (0.10)	−0.01 (0.04)	0.05 (0.07)
Conscientiousness	0.10 (0.09)	0.16 (0.09)^+^	−0.01 (0.04)	0.04 (0.07)
Agreeableness	−0.09 (0.09)	−0.02 (0.09)	−0.02 (0.04)	−0.12 (0.07)^+^
Gender × Neuro	0.03 (0.12)	0.22 (0.12)^+^	−0.17 (0.05)^**^	−0.01 (0.09)
Gender × Extra	−0.18 (0.12)	0.09 (0.12)	−0.05 (0.05)	0.08 (0.09)
Gender × Open	−0.01 (0.11)	0.07 (0.11)	−0.09 (0.05)^+^	−0.18 (0.08)^*^
Gender × Consc	0.04 (0.10)	0.03 (0.11)	−0.03 (0.05)	−0.06 (0.08)
Gender × Agree	0.04 (0.11)	0.04 (0.11)	−0.08 (0.05)	0.02 (0.08)
*R*-square Δ (*df* 1, 2)	0.01 (5, 777)	0.00 (5, 777)	0.02 (5, 777)^*^	0.07 (5, 777)

*BMI, body mass index. Δ, change. Numbers reflect unstandardized regression coefficients (with standard errors). Gender was uncentered (0 = men, 1 = women). Age and BMI were centered. Recruitment was uncentered (0 = Psychology classes; 1 = other). Neuroticism, Extraversion, Openness, Conscientiousness, and Agreeableness were standardized. The intercept reflects the average number of daily servings for men recruited from psychology classes at mean levels on the other predictor variables*.

+ p < 0.10;

* p < 0.05;

** p < 0.01;

*** *p < 0.001*.

Two other personality traits also predicted FV consumption. Unexpectedly, extraversion was a significant predictor of FV consumption, with young adults +1 *SD* above the mean in extraversion consuming 0.34 and 0.52 more daily servings of fruit (Sample 1 and 2, respectively) and 0.40 more daily servings of vegetables (Sample 2 only) compared to young adults −1 *SD* below the mean in extraversion. Conscientiousness also significantly predicted greater FV consumption in Sample 2 only. Young adults +1 *SD* above the mean in conscientiousness ate 0.22 more daily servings of fruit and 0.38 more daily servings of vegetables compared to participants −1 *SD* below the mean on conscientiousness.

Personality traits accounted for an additional 5–6% of the variance in FV consumption over and above the demographic factors, which only accounted for 2–3% of the variance in FV consumption. Women reported greater vegetable consumption than men across both studies (2.7 vs. 2.4 daily servings for women vs. men), and higher fruit consumption in Sample 1 only (1.9 vs. 1.5 daily servings for women vs. men). Older young adult age predicted less fruit and vegetable consumption in Sample 2 only. BMI was not related to FV consumption in either sample. Participants recruited from outside psychology classes in Sample 2 reported more FV consumption.

The patterns for unhealthy foods were different and less consistent than the patterns for FV consumption. In Sample 1, openness predicted less consumption of potato chips. In Sample 2, conscientiousness and agreeableness predicted less consumption of fries and candy.

Tables 2, 3 also present the results of the gender moderator analyses (Model 3). Gender did not moderate any of the relationships between personality traits and FV consumption in either sample. However, gender moderated the relationship between two personality traits and unhealthy food consumption in Sample 2. Neuroticism was associated with greater consumption of fries in men [*b*_(*SE*)_ = 0.14 (0.05), *t* = 3.08, *p* = 0.002], but not in women [*b*_(*SE*)_ = −0.03 (0.040), *t* = −1.04, *p* = 0.297], and conscientiousness was associated with less candy consumption in women [*b*_(*SE*)_ = −0.13 (0.05), *t* = −2.907, *p* = 0.004] but not in men [*b*_(*SE*)_ = 0.05 (0.06), *t* = 0.79, *p* = 0.429].

## Discussion

This study tested an important transition period of young adulthood and the role of personality traits in shaping healthy eating. Across two samples totaling 1,073 young adults, higher openness and extraversion were the most consistent personality predictors of greater FV consumption in this population. Conscientiousness also predicted FV consumption, but it was less consistent than the other two personality traits. Neither agreeableness nor neuroticism predicted FV consumption among young adults. These findings were specific to fruit and vegetables and mostly did not extend to unhealthy foods such as potato chips, French fries, and candy, although openness was associated with less consumption of potato chips in Sample 1.

The associations between the five combined personality traits and FV consumption reflected medium effect sizes (*R* Squared estimates ranging from 5 to 6%; which equate to Cohen's *d*s of 0.46 to 0.52, where 0.5 is a “medium” effect size; Cohen, 1988). These effect sizes are similar to a recent study showing that the combined Big Five personality traits predicted 7% of the variance in cardiometabolic risk over and above demographic covariates (Dermody et al., 2016). Expressing our findings in terms of serving sizes, people higher in openness, extraversion, or conscientiousness typically ate around 0.7 more daily servings of combined FV than people lower in these traits. This estimate reflects 0.3 or 0.4 more daily servings of fruit, which is equivalent to one-third or nearly half a banana or 10 to 14 grapes, and 0.4 more daily servings of vegetables, which is equivalent to five baby carrots or half a cup of raw greens, compared to people lower in each of these traits. Although these differences may seem small, daily differences can accumulate across the year, and lifetime. For example, the difference between someone high vs. low on openness would amount to over 250 more servings of FV each year (0.7 daily servings of FV × 365 days = 256 servings per year difference between high and low openness, assuming average levels on all other traits). However, these estimates are provided with one caveat: They are only an approximation and may not be exact given some imprecision in our measurement of FV (see Section Limitations).

These findings reinforce the importance of openness as a significant predictor of plant-food consumption in young adults, particularly vegetables. Openness is characterized by a desire for variety, a willingness to try new things, and higher intellect (DeYoung, 2010)—characteristics that could promote a more varied and healthy diet including plant-foods. Vegetables can be initially unpalatable and can evoke taste aversions in children, a behavioral characteristic that may have evolved to protect children from plant toxins (Cashdan, 1998). As a trait associated with exploration, openness may enable young adults to broaden their pallet, try new and unusual foods, and overcome taste aversions. Higher intellect among open young adults could also explain their lower consumption of several unhealthy foods (potato chips, fries, and candy). Intellect has been related to a lower consumption of junk food in previous research (Batty et al., 2007; Northstone et al., 2011). Overall, these patterns for openness are consistent with previous research showing that openness predicts both FV consumption in children (Vollrath et al., 2012), American college students (Raynor and Levine, 2009), adolescents (de Bruijn et al., 2005), and older adults (Tiainen et al., 2013). Our study adds to this evidence by replicating this association in the largest sample of young adults to date using real-time tracking of FV consumption and using control variables.

Extraversion also predicted higher FV consumption among our young adults, particularly in the larger Sample 2. If Sample 1 had been larger in size, the positive trend between extraversion and vegetable consumption might have been statistically significant. This pattern linking higher extraversion to FV consumption is consistent with some previous research showing that higher extraversion predicted greater FV consumption among Norwegian boys ages 6–12 (Vollrath et al., 2012) and greater vegetable consumption among older Finnish women (Tiainen et al., 2013). The findings for extraversion are not entirely consistent across studies and populations, but when associations are found, they tend to be in the direction observed here—higher extraversion predicting greater FV consumption. Although the mechanisms are not known, extraversion is associated with greater surgency and approach motivation (Depue and Collins, 1999), which could allow individuals to overcome natural taste aversions to eat more fruit and vegetables (Vollrath et al., 2012).

It is quite interesting that the two traits strongly associated with FV consumption in our two samples, openness and extraversion, together make up a higher order meta-trait called “plasticity,” which represents a “general tendency to explore and engage with possibilities” and includes a desire for novelty (DeYoung, 2010, p. 1170). Although plasticity has been linked to risky health behaviors in young men (e.g., drug use; DeYoung et al., 2008), our findings suggest a possible benefit of plasticity through greater FV consumption. Additional research is needed to understand the role of meta-traits in healthy eating. However, it would require a different analytic approach using structural equation modeling to model the shared variance rather than the independent variance as we did here. For example, a recent study by Dermody et al. (2016) found that the meta-trait of stability (agreeableness, conscientiousness, and lower neuroticism) predicted lower cardiometabolic risk (a composite measure of blood pressure, insulin resistance, BMI, and cholesterol), but they found no evidence that plasticity was linked to these outcomes. Only openness, but not extraversion, was independently related to lower cardiometabolic risk (Dermody et al., 2016).

Conscientiousness also predicted greater FV consumption, but less consistently than openness and extraversion. We found this association only in the larger sample. It is interesting that conscientiousness tends to be the single strongest predictor of other health behaviors such as alcohol use and smoking (Bogg and Roberts, 2004; Raynor and Levine, 2009; Turiano et al., 2015) but it is less consistently associated with FV consumption across studies. Although the previous study of young adults found a link between conscientiousness and greater FV consumption (Raynor and Levine, 2009), research on children and older adults has found fewer links between conscientiousness and FV consumption. It is possible that characteristics associated with conscientiousness such as discipline, while critical to a wider range of health behaviors, may not matter as consistently for plant-food consumption, which has more of a taste component. Characteristics associated with openness such as curiosity and exploration may matter more.

There were several gender differences in our data. The main difference was that young adult women ate more FV than young adult men. Women were closer to the “5 a day” target (4.5 or 4.7 daily servings of combined FV in Sample 1 and 2, respectively), whereas men were one serving away from this target (3.8 or 4.2 daily servings of FV in Sample 1 and 2, respectively). This gender difference in FV consumption is consistent with previous research (e.g., Emanuel et al., 2012). Men also ate more fries than women, but women ate more candy and chocolate than men. Importantly, gender did not moderate any of the associations between personality traits and FV consumption. This lack of moderation suggests that the associations between personality traits and FV consumption are generalizable across gender. In fact, gender only moderated two out of 40 relationships tested (5 personality traits x 4 foods x 2 samples)—the association between neuroticism and fries (in men, higher neuroticism corresponded with more fries consumption) and the association between conscientiousness and candy (in women, higher conscientiousness corresponded with less candy consumption). Given the large number of moderation tests performed, we do not put too much weight on these two findings.

### Implications

Our findings suggest that personality traits are important in establishing healthy habits in early adulthood. These traits—notably openness, extraversion, and conscientiousness—could set the stage for better health now and later in life through increased FV consumption. In the very long term, greater FV consumption could be a potential behavioral mechanism linking these personality traits to greater cardiovascular health and longevity (e.g., Taylor et al., 2009; Dermody et al., 2016). For example, openness was recently found to be a significant independent predictor of lower midlife cardiometabolic risk over and above the effects of the higher-order stability factor on reduced cardiometabolic risk (Dermody et al., 2016). And, whereas the higher-order stability factor was related to cardiovascular risk through intermediate pathways of inflammation, heart-rate variability, and exercise, none of these intermediate pathways explained the link between openness and cardiometabolic risk (Dermody et al., 2016). Our findings suggest that higher FV consumption could be an intermediary behavior linking openness to reduced cardiometabolic risk. We recommend including FV consumption in the potential list of mechanisms that could account for the relationship between personality and morbidity/mortality.

These results also help bridge the gap between adolescence and adulthood, providing support for the idea that young adulthood may be the time where personality influences on dietary patterns shift. The biggest difference from previous research on children and adolescents was that agreeableness was not associated with FV consumption, suggesting that among young adults, this trait is no longer as relevant for this health behavior. Young adulthood represents a critical transition period where people are moving out of their parental home and developing autonomy in their food choices. Consequently, being a more open person rather than a more agreeable person may influence their FV consumption to a greater extent as it is no longer necessary to be compliant with parental requests to eat these foods.

Our findings linking personality traits to FV consumption may have implications for promoting healthier dietary habits in the wider population. There has been some success at using interventions to shift personality traits associated with healthy lifestyle habits. For example, Magidson et al. (2014) presented a case study showing that engaging in 5 weeks of conscientious-like activities resulted in a reduction in substance use for a 45-year old man undergoing outpatient treatment (Magidson et al., 2014). If more openness promotes healthy dietary choices, interventions designed to increase openness should be beneficial. For example, one could design an intervention to increase openness toward FV by encouraging young adults to explore novel foods and try new plant-foods through a “Try it!” campaign. However, given that higher openness can be associated with higher drug use (e.g., marijuana, Terracciano et al., 2008), some caution would be in order. It would also be interesting to know whether interventions designed to increase intellect, a core feature of openness, would also increase FV consumption.

### Strengths and limitations

The strengths of this research included our computerized daily reporting of food across a 2- or 3-week period to better represent individuals' usual intake (Committee on Diet and Health Food and Nutrition Board, and Commission on Life Sciences National Research Council, 1989). We also used near-to-real-time daily diaries which reduced the reliance on memory recall. While some memory recall was required to report fruit and vegetables consumed that day and the previous night, this was less demanding than recalling over the course of a week or “typical” consumption. In addition, we had a relatively large sample size, particularly in Sample 2. The inclusion of a larger replication sample shows the importance of not relying on any single small study to draw conclusions. Lastly, we measured other unhealthy foods to show that personality traits predict higher consumption of fruits and vegetables *per se* and not just all foods.

Limitations included the cross-sectional design, so we cannot draw inferences about how these personality influences are changing developmentally. We also relied solely on self-reported FV consumption. Although participants reported their consumption in “near to real time” with clear serving size guidelines, there is still some unreliability in these reports. The rates of reported fruit and vegetable consumption were higher than those reported in other studies with young adults (Unüsan, 2004; see Dodd et al., 2010). It is possible that our participants' FV estimates were inflated due to biases in social desirability, which can occur when people report foods widely believed to be “healthy” such as fruit and vegetables (Hebert et al., 1995, 1997). The consumption estimates should be interpreted with that limitation in mind. Also, readers should be cautious about drawing precise conclusions about the serving size differences given the impreciseness in our outcome variables particularly at the high end of the scale which grouped “3 or more servings” in Sample 1, or “4 or more servings” in Sample 2. Thus, a score of 3 (or 4 in Sample 2) might mean one person ate three servings of vegetables that day, but another person ate six servings. Future research would benefit from open-ended reporting or a wider response scale. Another limitation was the smaller sample size of Sample 1 (*N* = 281). Although, we made up for this with the large size of Sample 2 (*N* = 792), some of the associations tested in Sample 1 might have been underpowered. Finally, we did not test how FV consumption was related to different facets, aspects, or meta-traits of these five personality variables. More detailed personality measures such as the 240-item Neuroticism Extraversion Openness Personality Inventory Revised (NEO-PI-R; Costa and McCrae, 1992) or the 100-item Big Five Aspect Scales (BFAS; DeYoung et al., 2007) would have provided finer resolution. The BFAS would have the added advantage of testing links between FV and openness and intellect separately. Given our findings for openness, it would be interesting to know how much of this association is being driven by the intellect aspect.

## Conclusion

Eat your fruits and vegetables. It turns out that some young adults are better at this than others. Young adults higher in openness and extraversion, and to some extent conscientiousness, ate more fruits and vegetables than their less open, less extraverted, and less conscientious peers. While the role of these traits varied across two samples, these three traits appeared to play significant roles, while agreeableness and neuroticism did not. These results bridge a gap in previous research between adolescence and adulthood, and suggest that young adults look more like adults in terms of how their personalities predict healthy habits. These results also suggest that FV consumption should be investigated as a health behavior linking personality traits like openness to greater physical and mental health in midlife and beyond.

## Ethics statement

This study was carried out in accordance with the recommendations of the University of Otago Human Ethics committee with written informed consent from all subjects. All subjects gave written informed consent in accordance with the Declaration of Helsinki. The protocol was approved by the University of Otago Human Ethics committee.

## Author contributions

TC conceived of the project and research question. LT, RK, JF, AR, and KB collected the data. TC and RN conducted the data analyses. TC, LT, RK, JF, AR, and KB wrote the paper. TC accepts full responsibility for the final content of the paper.

## Funding

The authors disclosed receipt of the following financial support for the research, authorship, and/or publication of this article: Preparation of this manuscript was supported by grant 12/709 from the New Zealand Health Research Council and a University of Otago Research Grant.

### Conflict of interest statement

The authors declare that the research was conducted in the absence of any commercial or financial relationships that could be construed as a potential conflict of interest. The reviewer EDC and the handling Editor declared their shared affiliation, and the handling Editor states that the process nevertheless met the standards of a fair and objective review.
