# Corrigendum: The Role of Personality Traits in Young Adult Fruit and Vegetable Consumption

**DOI:** 10.3389/fpsyg.2018.01597

**Published:** 2018-09-20

**Authors:** Tamlin S. Conner, Laura M. Thompson, Rachel L. Knight, Jayde A. M. Flett, Aimee C. Richardson, Kate L. Brookie

**Affiliations:** ^1^Department of Psychology, University of Otago, Dunedin, New Zealand; ^2^Department of Psychological Medicine, University of Auckland, Auckland, New Zealand

**Keywords:** personality, Mediterranean diet, health behaviors, daily diary methods, diet, young adult

In the original article, there was a mistake in Table 1 and Table 3 as published. The statistics for Openness and Conscientiousness were incorrect in Study 2 due to a coding error, which incorrectly labeled Openness as Conscientiousness and vice versa. The corrected Table 1 and Table 3 appear below. In addition corrections have been made in the following places:

**Table 1:** Participant characteristics and descriptive statistics for the two samples of young adults.

	**Mean**	***SD***	**Minimum**	**Maximum**	***n* (%)**
**SAMPLE 1**					
N					281
Male					128 (45.6%)
Female					153 (54.4%)
% European					83.6%
Age (years)	19.90	1.24	17.00	25.00	
BMI^a^	23.78	3.47	16.18	37.78	
Neuroticism	2.80	0.73	1.25	4.50	
Extraversion	3.51	0.50	1.58	4.83	
Openness	3.49	0.50	2.17	4.83	
Conscientiousness	3.32	0.60	1.75	4.83	
Agreeableness	3.59	0.47	2.17	4.75	
Fruit/day	1.70	1.08	0.00	5.73	
Vegetables/day	2.51	1.07	0.20	5.76	
Chips/day	0.45	0.57	0.00	5.77	
Cookies/day	0.40	0.42	0.00	2.22	
**SAMPLE 2**					
N					792
Male					217 (27.4%)
Female					575 (72.6%)
% European					77.5%
Age (years)	19.73	1.73	17.00	25.00	
BMI^b^	23.99	4.52	13.43	57.24	
Neuroticism	2.91	0.72	1.08	4.83	
Extraversion	3.51	0.52	1.50	4.83	
Openness	3.46	0.53	2.08	4.75	
Conscientiousness	3.49	0.61	1.25	4.92	
Agreeableness	3.74	0.50	2.25	4.83	
Fruit/day	2.08	1.29	0.00	7.00	
Vegetables/day	2.76	1.38	0.00	7.70	
Fries/day	0.56	0.60	0.00	4.08	
Candy/day	1.27	0.94	0.00	6.30	

BMI, body mass index.

a BMI computed from self-reported height and weight.

b *BMI computed from objectively measured height and weight. Food consumption variables expressed in standard serving sizes*.

**Table 3:** Results of hierarchical regression analyses predicting young adults' fruit and vegetable consumption and unhealthy foods in Sample 2 (*N* = 792).

	**Fruit**	**Vegetables**	**Fries**	**Candy**
**MODEL 1**				
Intercept	1.83 (0.10)^***^	2.34 (0.11)^***^	0.82 (0.05)^***^	0.96 (0.07)^***^
Gender	0.15 (0.10)	0.36 (0.11)^**^	−0.27 (0.05)^***^	0.36 (0.07)^***^
Age	−0.08 (0.03)^**^	−0.06 (0.03)^*^	−0.01 (0.01)	−0.02 (0.02)
BMI	−0.01 (0.01)	−0.01 (0.01)	0.00 (0.00)	0.01 (0.01)
Recruitment	0.34 (0.10)^**^	0.38 (0.11)^***^	−0.16 (0.05)^**^	0.13 (0.07)^*^
R-square Δ (df 1,2)	0.02 (4,787)^**^	0.03 (4,787)^***^	0.06 (4,787)^***^	0.03 (4,787)^***^
**MODEL 2**				
Intercept	1.84 (0.10)^***^	2.37 (0.11)^***^	0.79 (0.05)^***^	0.90 (0.08)^***^
Gender	0.14 (0.11)	0.33 (0.11)^**^	−0.23 (0.05)^***^	0.42 (0.08)^***^
Age	−0.09 (0.03)^**^	−0.07 (0.03)^*^	−0.01 (0.01)	−0.02 (0.02)
BMI	0.00 (0.01)	0.00 (0.01)	0.00 (0.00)	0.00 (0.01)
Recruitment	0.33 (0.10)^**^	0.35 (0.10)^**^	−0.14 (0.05)^**^	0.15 (0.07)^*^
Neuroticism	0.02 (0.05)	−0.02 (0.06)	0.02 (0.02)	0.01 (0.04)
Extraversion	0.26 (0.05)^***^	0.20 (0.05)^***^	0.04 (0.02)^*^	0.02 (0.04)
Openness	0.13 (0.05)^**^	0.18 (0.05)^***^	−0.03 (0.02)	0.00 (0.03)
Conscientiousness	0.11 (0.05)^*^	0.19 (0.05)^***^	−0.08 (0.02)^***^	−0.08 (0.04)^*^
Agreeableness	−0.06 (0.05)	0.00 (0.05)	−0.08 (0.02)^**^	−0.12 (0.04)^**^
R–square Δ (df 1,2)	0.05 (5,782)^***^	0.06 (5,782)^***^	0.03 (5,782)^***^	0.02 (5,782)^**^
**MODEL 3**				
Intercept	1.84 (0.11)^***^	2.31 (0.12)^***^	0.85 (0.05)^***^	0.91 (0.08)^***^
Gender	0.14 (0.11)	0.39 (0.12)^**^	−0.29 (0.05)^***^	0.41 (0.08)^***^
Age	−0.09 (0.03)^**^	−0.07 (0.03)^*^	−0.01 (0.01)	−0.02 (0.02)
BMI	−0.01 (0.01)	0.00 (0.01)	0.00 (0.00)	0.00 (0.01)
Recruitment	0.33 (0.10)^**^	0.34 (0.10)^**^	−0.13 (0.05)^**^	0.16 (0.07)^*^
Neuroticism	0.00 (0.10)	−0.18 (0.10)^*^	0.14 (0.05)^**^	0.02 (0.07)
Extraversion	0.38 (0.10)^***^	0.13 (0.10)	0.08 (0.05)^*^	−0.04 (0.07)
Openness	0.10 (0.09)	0.16 (0.09)^*^	−0.01 (0.04)	0.04 (0.07)
Conscientiousness	0.10 (0.09)	0.14 (0.10)	−0.01 (0.04)	0.05 (0.07)
Agreeableness	−0.09 (0.09)	−0.02 (0.09)	−0.02 (0.04)	−0.12 (0.07)^*^
Gender x Neuro	0.03 (0.12)	0.22 (0.12)^*^	−0.17 (0.05)^**^	−0.01 (0.09)
Gender x Extra	−0.18 (0.12)	0.09 (0.12)	−0.05 (0.05)	0.08 (0.09)
Gender x Open	0.04 (0.10)	0.03 (0.11)	−0.03 (0.05)	−0.06 (0.08)
Gender x Consc	−0.01 (0.11)	0.07 (0.11)	−0.09 (0.05)^*^	−0.18 (0.08)^*^
Gender x Agree	0.04 (0.11)	0.04 (0.11)	−0.08 (0.05)	0.02 (0.08)
R-square Δ (df 1,2)	0.01 (5,777)	0.00 (5,777)	0.02 (5,777)^*^	0.07 (5,777)

BMI, body mass index. Δ, change. Numbers reflect unstandardized regression coefficients (with standard errors). Gender was uncentered (0 = men, 1 = women). Age and BMI were centered. Recruitment was uncentered (0 = Psychology classes; 1 = other). Neuroticism, Extraversion, Openness, Conscientiousness, and Agreeableness were standardized. The intercept reflects the average number of daily servings for men recruited from psychology classes at mean levels on the other predictor variables.

* p < 0.10;

* p < 0.05;

** p < 0.01;

*** *p < 0.001*.

Methods, Data Preparation and Analysis, Paragraph 1
Results, Regression Results, Paragraphs 1 and 2
Results, Regression Results, Paragraphs 4 and 5
Discussion, Paragraph 1
Discussion, Paragraph 7

**Methods, Data Preparation and Analysis, Paragraph 1**

Excluded participants in Sample 2 were more likely to be male [χ2_(1, *N*=827)_ = 7.41, *p* = 0.007], and score lower in conscientiousness [3.1 vs. 3.5; *t*_(825)_ = −3.58, *p* < 0.001] and agreeableness [3.5 vs. 3.7; *t*_(825)_ = −3.35, *p* = 0.001] than included participants.

**Results, Regression Results, Paragraphs 1 and 2**

In terms of serving sizes, young adults one standard deviation above the mean (+ 1 *SD*) in openness ate 0.26 and 0.26 more daily servings of fruit (Sample 1 and 2, respectively) and 0.38 and 0.36 more daily servings of vegetables (Sample 1 and 2, respectively) compared to participants one standard deviation below the mean (−1 *SD*) in openness.

Young adults + 1 *SD* above the mean in conscientiousness ate 0.22 more daily servings of fruit and 0.38 more daily servings of vegetables compared to participants −1 *SD* below the mean on conscientiousness.

**Results, Regression Results, Paragraphs 4 and 5**

The patterns for unhealthy foods were different and less consistent than the patterns for FV consumption. In Sample 1, openness predicted less consumption of potato chips. In Sample 2, conscientiousness and agreeableness predicted less consumption of fries and candy.

Neuroticism was associated with greater consumption of fries in men [*b*_(*SE*)_ = 0.14 (0.05), *t* = 3.08, *p* = 0.002], but not in women [*b*_(*SE*)_ = −0.03 (0.040), *t* = −1.04, *p* = 0.297], and conscientiousness was associated with less candy consumption in women [*b*_(*SE*)_ = −0.13 (0.05), *t* = −2.907, *p* = 0.004] but not in men [*b*_(*SE*)_ = 0.05 (0.06), *t* = 0.79, *p* = 0.429].

**Discussion, Paragraph 1**

These findings were specific to fruit and vegetables and mostly did not extend to unhealthy foods such as potato chips, French fries, and candy, although openness was associated with less consumption of potato chips in Sample 1.

**Discussion, Paragraph 7**

In fact, gender only moderated two out of 40 relationships tested (5 personality traits x 4 foods x 2 samples)—the association between neuroticism and fries (in men, higher neuroticism corresponded with more fries consumption) and the association between conscientiousness and candy (in women, higher conscientiousness corresponded with less candy consumption). Given the large number of moderation tests performed, we do not put too much weight on these two findings.

The authors apologize for this error and state that this does not change the scientific conclusions of the article in any substantive way.

The original article has been updated.

## Conflict of interest statement

The authors declare that the research was conducted in the absence of any commercial or financial relationships that could be construed as a potential conflict of interest.

